# Gel performance in rheology and profile control under low-frequency vibration: coupling application of physical and chemical EOR techniques

**DOI:** 10.1007/s13202-016-0267-1

**Published:** 2016-06-29

**Authors:** Li Ming Zheng, Chun Sheng Pu, Jing Liu, Bo Ma, Nasir Khan

**Affiliations:** 1 College of Petroleum Engineering, China University of Petroleum (Eastern China), No. 66 Yangtze River West Road, Huang Dao District, Qingdao, 266580 China; 2 State Key Laboratory of Heavy Oil, Qingdao, China; 3 The Seventh Oil Production Plant of Changqing Oil Field, Xi’an, China

**Keywords:** Flowing gel, Low-frequency vibration, Rheological change, Gel cross-linking stages, Gel injection

## Abstract

Flowing gel plugging and low-frequency vibration oil extraction technology have been widely applied in low-permeability formation. High probability of overlapping in action spheres of two technologies might lead to poor operating efficiency during gel injection. Study on flowing gel rheological properties under low-frequency vibration was essential, which was carried out indoor with viscosity measurement. Potential dynamic mechanisms were analyzed for the rheological variation. Under low-frequency vibration, gel rheological properties were found to be obviously influenced, with vibration delaying gel cross-linking in induction period, causing a two-stage gel viscosity change in acceleration period, and decreasing gel strength in stable period. Surface of gel system under vibration presented different fluctuating phenomenon from initial harmonic vibrating to heterogeneous fluctuating (droplet separation might appear) to final harmonic vibrating again. Dynamic displacement in unconsolidated sand pack revealed that low-frequency vibration during gel injection might be a measure to achieve deep profile control, with the gel injection depth increased by 65.8 % compared with the vibration-free sample. At last, suggestions for field test were given in the paper to achieve lower injection friction and better gel plugging efficiency.

## Introduction

At present, in a large number of special oil or gas fields, low-frequency vibration oil extraction technology and flowing gel plugging technology have been widely used to improve the overall development efficiency of reservoirs (Kang and Pu 2007). Low-frequency vibration oil extraction technology, a physical enhancement technology, transmits powerful vibration energy such as harmonic wave or pulse wave on the layer directly or indirectly. Through the wave spreading in the formation, the reservoir sand and fluid fluctuate. It leads to various physical and chemical changes, which improve the reservoir seepage and performance of oil and water wells. Flowing gel plugging technology is widely used in oil or water wells to plug water channeling and improve flooding in vertical or radial heterogeneous formation so as to increase the displacement of oil trapped in pores and reservoir recovery. Flowing gel used in low-permeability field trials could be divided into chromium (III) gel and aluminum gel and so on, according to the main chemical elements cross-linked. It is also divided into organic gel and inorganic gel, according to the molecule group property connected with the cross-linking element.

Due to the low attenuation of low frequency wave propagating in the porous media, the low-frequency vibration oil extraction technology has a large effective action radius when it is applied in the oilfield (White 1975). Therefore, there is a high possibility of overlapping in sphere of action of two technologies. Sometimes, the flowing gel injection area may be covered in the range of vibration oil recovery technique, which may cause certain effect on gelling process, ultimate gel strength and gel plugging efficiency. The whole gel injection process combines the ground mix, pipe injection, pushed into the oil layer, flowing in the pore throats, and achieving the stable gel strength finally. In the gel accelerating stage, the gel strength as well as the injecting pressure and friction factor may sharply increase because of the violent disturbance, which could bring severe problems to gel displacement. The construction success rate may be harmed thereby (Junowicz et al. 1972; Broseta et al. 2000), whereas low-frequency vibration also has the functions of increasing internal fluid contact, shear thinning, and improving the seepage velocity inside the porous media (Ma et al. 1996). Considering the above factors, the composite action mechanisms of two technologies become much complex. Research on change of flowing gel rheological property under low-frequency vibration is important for optimizing the profile control and water plugging effects.

Mechanisms about gel displacement and low-frequency vibrating oil production had been studied separately in lots of papers, but researches about low-frequency vibrating on the flowing gel rheological variation and gel flow in underground formation were barely found. Under the background of vacancy acknowledgement toward the composite action mechanisms, an investigation on change of flowing gel rheological property under low-frequency vibration is carried out. Gel rheological properties analysis was done with viscosity measurement (Huang et al. 2001; Wang et al. 1995), gel surface observation, and displacement in artificial core. Potential micro-dynamic causes were predicted for the rheological properties variation. Suggestions for the above two technologies field composite application were provided at last.

## Experimental apparatus and procedures

### Apparatus and materials

The experimental apparatus included the low-frequency vibrating oil extraction equipment (designed by the Physical & Eco-Chemical Technology and Engineering Center for Complex Hydrocarbon Reservoir Stimulation), Brookfield DV-III type rotational viscometer, thermostat water bath, thermometer, and unconsolidated sand pack, etc. The low-frequency vibrating oil extraction equipment was composed of vibrostand (including the vibration table and pedestal), vibration exciter, power amplifier, control cabinet and heat sink.

Laboratory experiment materials included gel cross-linking agent (CrCl_3_·6H_2_O), polymer (HPAM, molecular weight of 10 million) and artificial reservoir water (according the typical reservoir water properties in Chang 6 reservoir in Ordos basin, salinity 40,000 mg/L and electrolytes ratio as NaCl/CaCl_2_/MgCl_2_ = 0.7:0.12:0.18).

### Experimental procedure

#### Rheology under vibration

**Fig. 1:**
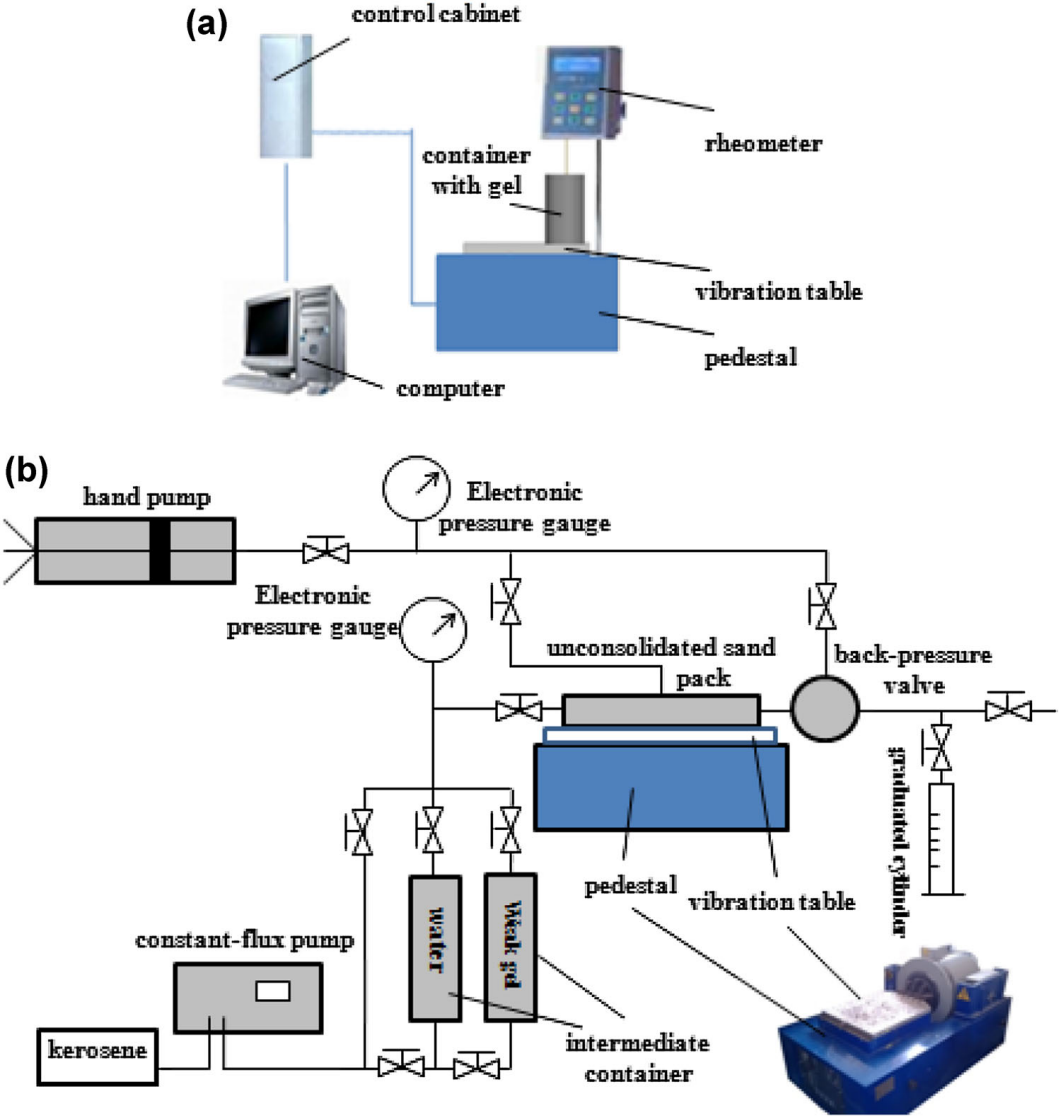
Indoor experiment apparatus for low-frequency vibrating. **a** Gel viscosity measurement under low-frequency vibration. **b** Gel displacement under low-frequency vibration

Chromium gels were configured in the beaker and tested for gel rheology change under low-frequency vibration as in Fig. 1a. Gel viscosity, increasing gradually with time without vibration, would be measured at regular intervals with the Brookfield viscometer until the stable stage value. Because the thermostat water bath could not be fixed on vibrating device in case of fluctuation damage, the beaker containing gel would not be heated up when on the vibrating device. To ensure the consistency in ambient temperature during gel strength measurement, the beakers were repeatedly putting in the water bath for a fixed time period and taking them out to measure the viscosity in the atmospheric environment or on the vibrating device for another fixed time interval. Each time the beaker was taken out from the water bath, the viscosity had been measured instantly at the beginning and before putting the beaker back into the water bath again. In addition, two samples, one under vibration and the other kept in the atmosphere, had been configured each time to minimize the errors caused by the environmental temperature variation as far as possible.

For gel rheological property research in different gel cross-linking phases under vibration, the beaker was fixed on the vibrating table only at specific gelling process and kept static in the atmosphere at other stages. The vibration exciter was operated at a given vibrating frequency 18 Hz (close to the natural frequency of the oil layer in chosen study region) and acceleration 0.4 m/s^2^. The experimental ambient temperature was 20–25 °C.

#### Gel displacement under vibration

Three experiments were conducted with unconsolidated sand pack model to study the gel performance in water plugging under low-frequency vibration as in Fig. 1b, symbolizing the coupling application of low-frequency vibration oil extraction technology and gel displacement technology. Optimization on parameters which influenced gel injection was not involved. Experimental conditions included gel injection without vibration (as a control group), vibration after gel injection and cross-linking (analyzing the influence of vibration on gelling in stability stage), and gel injection with vibration simultaneously (analyzing the influence of vibration on gelling in induction stage and accelerating stage). Vibration acceleration and frequency were kept with 0.4 m/s^2^ and 18 Hz, respectively.

In the control group (I), after the measurements of pressure drop and rock permeability to water, gel of 0.2 PV and subsequent water slug of 0.2 PV were injected in the core successively, and 40 h for complete gel cross-linking in thermostat was needed. Then, the pressure drop and permeability to water of the core would be measured again. To analyze the influence of vibration on gelling in induction stage and accelerating stage, the unconsolidated sand pack (II) was fixed on the vibrating device before gel injection. Once the vibrating device was set on, gel was injected. The vibration lasted for 25 min, which was enough for gel and subsequent water injection. To analyze the influence of vibration on gelling in stability stage, time of thirty-nine hours and forty-five minutes was set for gel cross-linking in the thermostat, and then the core (III) was taken out and fixed on the vibrating device for fluctuation treatment which also lasted for 25 min. Other steps were same as above.

## Experimental results

### Gelling process

 By measuring viscosity change of normal gel without vibration, the gelling process was divided into three stages: stage I, stage II and stage III (Duan 2002; Cameron 1993; Meynie et al. 2004; Zhang et al. 2007). Formation of internal gel cross-linking network could be abstracted as a growth from two-dimensional molecular group in early stage to later three-dimensional congregation to the final stable gel system.

- Stage I: Gel cross-linking induction stage. In this stage, the viscosity remained little and changed slowly. Intramolecular cross-linking appeared gradually after gel cross-linking agent and polymer were mixed. Many single-molecule clusters, which were polynuclear olation complex ions actually and looked like tight threads, were generated later because of the intramolecular contraction. Though the number of clusters was large, gel system apparent viscosity was barely influenced.
- Stage II: Gel viscosity accelerating increase stage. In this stage, gel viscosity rose rapidly from initial value to the maximum value. Intermolecular cross-linking between the polynuclear olation complex ions increased in the gel system, and three-dimensional network structure was formed. Different from stage I, though the number of new intermolecular junction point per unit time was little, remarkable increase was observed on the apparent viscosity of gel.
- Stage III: Gel viscosity stable stage. During this period, gel viscosity remained in a steady range and reached the strength to plug water channeling. While there were still a few amounts of single-molecule clusters inside the gel, three-dimensional network structure had already reached stability through intermolecular cross-linking. It could maintain for a certain period.

Gel in this experiment was originally used for water plugging in low-permeability reservoir with micro-fractures, so the gel strength was relatively small. In view of the different viscosity characteristics in gelling process, the following analyzed the gel rheological change in different gelling stages under vibration successively.

### Viscosity analysis in different gelling stage under vibration

In different gelling stages, influences of low-frequency vibrating on gel viscosity variation were studied indoor with keeping one sample static. An explanation was conjectured about the micro-interaction mechanisms between vibration and gel.

#### Influence in induction stage

**Fig. 2:**
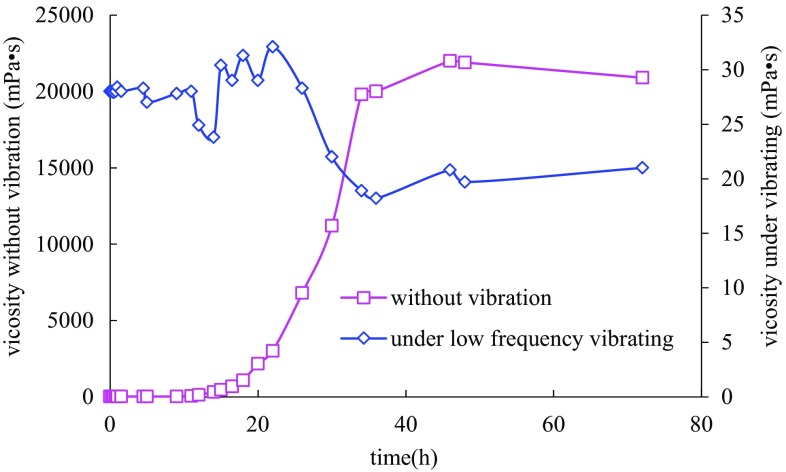
Sol process comparison chart of normal gel and gel under vibration in stage I for 24 h

Gel rheological change in induction stage under vibration for 24 h, which was far more than the gelation time, was studied firstly (Fig. 2). Through analysis about the viscosity versus time in this experiment, gel was found to be completely destroyed under longtime vibration. Components for gel cross-linking could not aggregate effectively and cross-link successively inside the mixture. Investigating the reason causing the phenomenon above, complexation between polymer molecules and cross-linking agents was reckoned to be around the main element-chromium. Molecule contraction caused by intramolecular force led to the formation of a large number of small and compact monomolecular clusters or other stable micelles after long-time low-frequency vibration (Duan 2002; Dukhin 2007). These stable monomolecular groups with micron size as well as relatively increased dispersion distance resulted in unsuccessful intermolecular cross-linking in gel accelerating stage, not to mention the 3D network structure. In addition, the little acting force between monomolecular groups also had made the failure of gelling even after vibrating was stopped.

The viscosity in Fig. 2 was also found to increase firstly and decrease later. The viscosity measured was known to reflect the value of shear force between the micelles indirectly (Walkenstrom et al. 1998). The viscosity increasing showed that vibration had promoted the mix between internal gelling agents. Shear force between the monomolecular groups formed was larger than that of polymer molecules. The viscosity decreasing, however, reflected the shear damage to polymer molecule chain under long-time vibration and the reduction in polymer chain molecular weight and intermolecular forces.

**Fig. 3:**
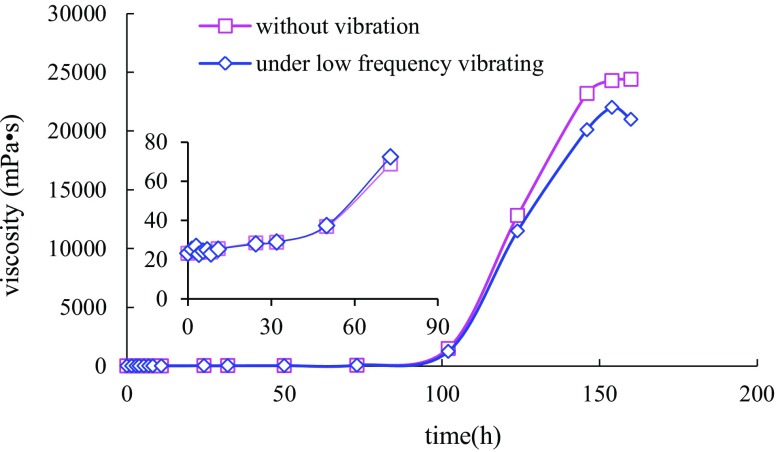
Sol process comparison chart of normal gel and gel under vibration in stage I for 9 h

After the experiment about longtime vibration to gelling, the question about “How the gelling process would change when reducing the time for vibration appropriately during the induction stage” was answered. With vibration for 9 h (less than the gelation time), a new result was got as Fig. 3. Due to experimental environment temperature change and viscosity measurement error, the viscosities before and after gelling as well as the whole gelling time were different from the above experiment result. The corresponding error had been eliminated by measuring new gel samples in a static state at the same time. After vibration for 9 h, gel viscosity had a similar rising tendency as the one keeping static. The initial gel viscosity was slightly higher, and the ultimate viscosity was 15 % lower than that of normal sample. Initial oscillations at the induction stage had aggravated the contact between the polymer and cross-linking agent, but reduced the cross-linking time and amount of junction points between two-dimensional molecule clusters to a certain extent at the same time, which led to the decline of reticular structure stability and gel strength.

The above experiments showed that induction stage was a period for effective contact of cross-linking agent and polymer, and for the formation of two-dimensional molecule structure. Additionally, effective contact between the agents during the induction period and cross-link between junction points during the early accelerating period could be essential in forming the three-dimensional network structure of enough strength to plug fluid channeling in porous media.

#### Influence in accelerating stage

**Fig. 4:**
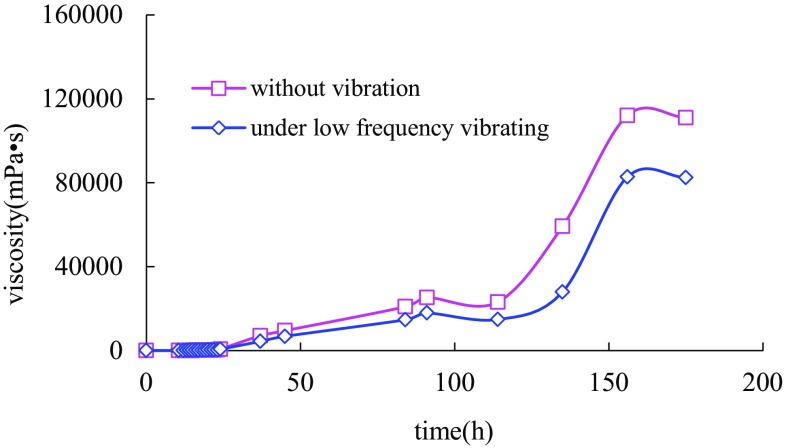
Sol process comparison chart of normal gel and gel under vibration in stage II for 12 h

After gel configuration, gel viscosity measurement started following the above procedures. The gelling condition of two samples was kept the same during the induction period. Only in acceleration stage, one sample was put on the vibrating table. The gel viscosity versus time was shown in Fig. 4. At the early stage, gel viscosity under vibration in accelerating stage was found to be of little difference from the static sample. But at the later stage of gelling process, the gelling velocity gradually slowed down after vibration for a certain time and the final gel strength was 25 % less than the static sample. Considering the viscosity change in Fig. 4, it was inferred that disturbance to gelling process in acceleration period had a larger influence to the final gel strength than that in induction period.

Two-dimensional molecular structures were known to cross-link and form three-dimensional network structure in gel accelerating stage. With the interference of low-frequency vibration, number of junction points between two-dimensional structures decreased obviously, leading to a reduction in interaction forces between three-dimensional network structures and eventual decline of the gel viscosity. It could also be found that the viscosity variation under low-frequency vibration or not had a similar tendency. It presented that low-frequency vibration not only affected the junction point number forming three-dimensional network structures, but also led to the retardation in gelling process.

#### Influence in stability stage

For the research about vibration on gel viscosity in stability stage, the gel configured was divided into two halves after the static value of apparent viscosity came to the maximum, one group staying continually in the air and the other putting on the vibrating table. The gel viscosities of two samples were measured following the similar steps.

**Fig. 5:**
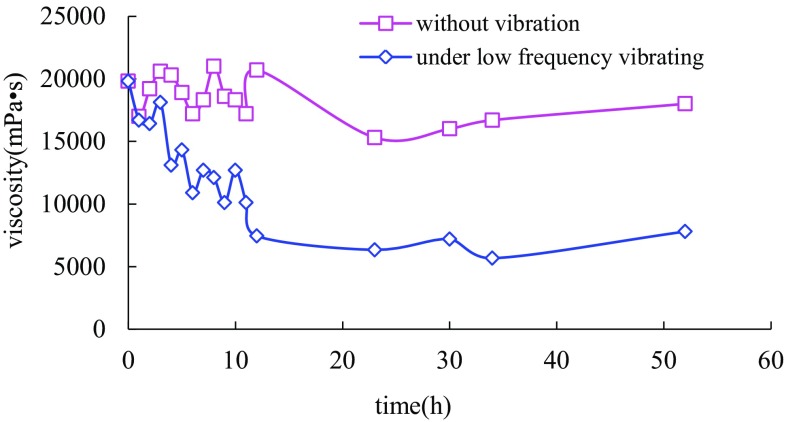
Sol process comparison chart of normal gel and gel under vibration in stage III for 12 h

Gel viscosity under vibration for 12 h in stability stage (Fig. 5) had declined compared with the static sample. After the viscosity achieving the maximum value, a certain rate of descent kept once fluctuating being exerted. The vibrating time was linear with the rate of viscosity descent. The average gradient was close to −1000 mPa s h^−1^. When vibration stopped at 12 h, gel viscosity had rebounded slightly and remained the same as a whole. It was inferred that three-dimensional molecular structure had reached a steady state and influence of low-frequency vibration on gel viscosity had the characteristic of irreversibility in stability stage.

### Permeability and resistance factor in dynamic displacement

Through the experiments on viscosity evaluation under vibration, it was known that low-frequency vibration had an obvious influence in gelling process. The viscosity change in three stages demonstrated different mechanisms in agents contact and cross-linking. To investigate the influence of vibration on dynamic gel plugging, three displacements were conducted as in section “Experimental procedure.” Results about the permeability and resistance factor under vibration or not are shown in Table 1.

**Table 1:** Comparison about gel performance in water plugging of three samples

Core number	I The control group	II Vibration after gel cross-linking	III Vibrating in gel injection
Permeability before gel injection/10^−3^ μm^2^	129.28	135.37	153.56
Permeability after gel injection/10^−3^ μm^2^	3.85	4.75	3.28
Plugging ratio/%	97.02	96.33	98.59
Breakthrough pressure/MPa m^−1^	4.39	3.55	5.06

As shown in Table 1, vibration after gel cross-linking had decreased the pressure drop for water breakthrough and the plugging efficiency. While vibration in gel injection had an opposite and favorable performance, which indicated that an operation of vibrating simultaneously with gel injection was advantageous to the gel plugging. Experimental analysis with resistance factor change had also proved above phenomenon. Ultimate values of resistance factor in three samples were 70 (vibrating while gel injection), 40 (without vibration) and 25 (vibrating after gel cross-linking), respectively.

The differences in gel properties and flooding depth under different vibrating time might account for above results. Gel strength, under vibration after gel cross-linking, was reduced obviously, which had caused decline in plugging ratio and resistance factor in the core. The increased matrix permeability and the looser cementation under vibration might also play a role in reducing the plugging effect. Gelation change under short-time vibration in induction stage and accelerating stage had proved that gel strength was scarcely influenced when gel was injected under vibration. About the increase in plugging ratio and resistance factor, another factor, the gel injection depth, might play a main role. As mentioned in introduction, low-frequency vibration could increase the fluid flow in porous media, so gel was easy to be impulsively displaced forward when its viscosity was relatively much low during the gel injection process. The increase in gel injection depth enabled the decrease in overall permeability and degree of water channeling.

## Discussions

Preliminary analysis of gel performance in dynamic displacement suggested that vibration while gel injection would be advantageous to the sweep efficiency improvement in extended water flooding, and it might be a new prospect for the exhausted traditional EOR or IOR techniques. It is not limited to low-permeability or heterogeneous reservoirs, and high water cut reservoirs might also work. Therefore, optimization of working parameters should be further conducted. Due to the low cost of low-frequency vibration oil extraction technology, a trial in low-permeability or high water cut reservoirs is worth of identifying the indoor result.

### Injection depth of gel under vibration

To explore the influence of vibration on gel plugging, the parameter of gel injection depth was calculated to represent the improvement of gel performance. Mathematical model of weak gel in-depth flooding (Zhang et al. 2007) was simplified as Eqs. (1)–(4) (Brenner et al. 2013).

1 $$\frac{\partial }{\partial t}\left( {\phi S_{0} } \right) = \nabla \left[ {\lambda_{0} \left( {\nabla P_{0} - \gamma_{0} \nabla D} \right)} \right] + q_{0} \delta$$

2 $$\frac{\partial }{\partial t}\left[ {\phi_{m} \left( {\chi_{m} S_{w} + C_{rm} } \right)} \right] = \frac{\partial }{\partial x}\left[ {\frac{{x_{m} \lambda_{m} \left( {\nabla P_{w} - \gamma_{w} \nabla D} \right)}}{{R_{k} }}} \right] + q_{m} \delta$$

3 $$P_{cow} = P_{0} - P_{w} = P_{c} \left( {S_{w} ,\sigma_{wo} } \right)$$

4 $$S_{o} + S_{w} = 1$$

where *t* is the time, s; *C*_*m*_ is the adsorption and retention volume of composition *m* in water phase, cm^3^; *ϕ* is the total porosity; *ϕ*_*m*_ is the porosity of composition *m*in water phase; *δ* is the Kronecker delta; *x*_*m*_ is the concentration of composition *m* in water phase, cm^3^/cm^3^; *q*_*m*_ is the injection volume of composition *m*, cm^3^; *S*_*w*_ is the water saturation; *S*_*o*_ is the oil saturation; *λ*_*m*_ is the mobility of composition *m*in water phase, μm^2^/mPa·s; *λ*_*o*_ is the oil mobility, μm^2^/mPa s; *D* is the depth from a certain base level, cm; *γ*_*w*_ is the weight of water phase; *P*_*cow*_ is the oil–water capillary pressure, 10^−1^ MPa; *P*_*c*_ is the capillary pressure function; *σ*_*wo*_ is the oil/water interfacial tension, N/m.

After simplification, a correlation was discovered between resistance factor and gel injection depth as Eq. (5). The resistance factor *R*_*k*_ in one-dimensional weak gel in-depth flooding model varied inversely with the product of concentration, water mobility, weight of water, and gel injection depth.

5 $$R_{k} \propto - x_{m} \lambda_{w} \gamma_{w} \nabla D$$

Assuming a homogeneous concentration and permeability was distributed in the gel injected area, so the gel injection depth was only changed. Ratio of resistance factors between the sample without vibration and that with gel injection and vibration simultaneously was approximately equal to the ratio of gel injection depth in two samples.

According to above derivation, gel injection depth was increased by 65.8 % under vibration when gel was injected compared with that without vibration, which indicated that low-frequency vibration in gel induction stage and accelerating stage might be an effective measure to achieve deep profile control in gel flooding.

### Guidance to field gel injection

Through research of gel rheological change under low-frequency vibration, vibration for a longtime in induction stage was found to be disadvantageous for forming the gel used in water channel plugging, but short-term vibrating could delay cross-linking and reduce resistance in near wellbore zone gel injection. Therefore, in field gel injection, formation with gel injected of induction stage should not be interfered with longtime low-frequency vibration.

At the early acceleration stage, low-frequency vibration might improve or reduce the gel viscosity. That effect depended on two aspects of the improved agents mix and shear dilution by vibration. At the late acceleration stage, viscosity under low-frequency vibration was lower than that of static gel. Therefore, optimization and adjustment of vibrating parameters was to be explored in field test to achieve lower gel injection resistance and delayed gel cross-linking so as to achieve deeper strata displacement and better plugging effect (Ariadji 2005).

In stability stage, gel viscosity under vibration was found to decrease linearly with time. Hence, vibration should be avoided as far as possible in the gel displacement work area, or stopped for a short period of time after gel viscosity got to the maximum value in order to weaken the damage to ultimate gel strength in field test.

To improve the oil displacement efficiency in composite application of low-frequency vibration oil extraction technology and gel plugging, it is necessary to further explore the dynamic mechanisms of two technologies, optimize experimental vibrating parameters, and carry out physical or numerical simulations for better gel plugging effect in porous media.

### Fluid surface vibrating

In gelling process of three stages, state of gel changed from a type close to Newton fluid in induction period to a type near solid in stability period. Gel rheological property going by the transition of “mobile, viscous, plastic” of fluid toward “elastic–plastic” of the solid. Gel had a feature of increased elasticity gradually and became a system with viscoelasticity (Prud’homme et al. 1984; Wang and Zhang 2003; Brenner et al. 2013).

**Fig. 6:**
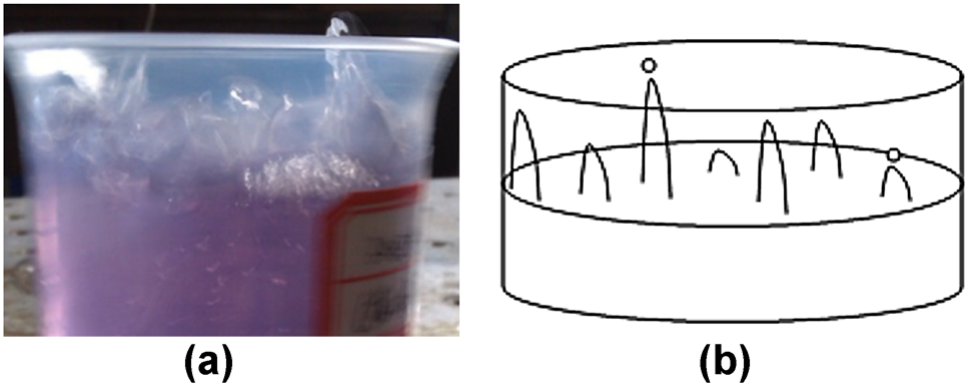
Vibrating phenomenon on fluid surface at accelerating stage. **a** Real photo of the surface vibrating. **b** Schematic diagram of surface vibrating with droplet separated

According to the change above, fluctuation of gel surface in induction period resembled the homogenous harmonic motion in quasi-Newton fluid, with each point having the similar characteristic of motion or homogeneity. In acceleration stage, due to the gel transitional change from plasticity to elasticity, obviously non-uniform fluctuating phenomenon appeared, with vibration amplitudes in some points greater than the others (Fig. 6) and even droplets separated from the top of the column (Fig. 6b) when in the down stroke. Figure 6a was the real photo of the surface vibrating to express clearly the phenomenon of inhomogeneous fluctuation. Elastoplasticity of gel increased as gel strength became stabilized gradually. Meanwhile, gel rheological property as well as the fluctuation in each part tended to the same again. Harmonic motion reappeared without droplets separation occurring. The fluctuation amplitude might be larger or smaller sometimes than that of the fluid surface in induction period, which was determined by the influences of vibration to gel elasticity and viscosity.

## Conclusions

Rheological property change of flowing gel under low-frequency vibration was studied with viscosity measurement and fluid surface fluctuating observation. The results obtained are summarized as following.

1. Low-frequency vibration oil extraction technology had different influence on flowing gel rheological properties in different gelling stages. Low-frequency vibration could delay cross-linking in induction stage, cause viscosity change of two phases in accelerating stage, and reduce the gel ultimate strength linearly with time in stability stage.
2. With gel viscoelasticity increasing, fluctuating on fluid surface went through the changes from homogenous harmonic motion to non-uniform vibration (droplets separation might appear) to homogenous harmonic motion again.
3. Various construction schemes should be made in the field application of low-frequency vibration oil extraction technology and gel plugging with different gelling stages to reduce the injection friction and improve the gel displacement effect.

## References

[CR1] Ariadji T (2005) Effect of vibration on rock and fluid properties: on seeking the vibroseismic technology mechanisms. SPE93112

[CR2] Brenner Tom, Matsukawa Shingo, Nishinari Katsuyoshi (2013). Failure in a soft gel: delayed failure and the dynamic yield stress. J Nonnewton Fluid Mech.

[CR3] Broseta D, Marquer O, Alain Z (2000). Shear effects on polyacrylamide/chromium (III) acetate gelation. SPE Reserv Eval Eng.

[CR4] Cameron JR (1993). Rheological modeling and scale-up of a delayed-crosslinked gel in nonhomogeneous flow. SPE Prod Facil.

[CR5] Duan Hongdong (2002). Study on the cross linking mechanism and dynamics of gel ation of partially hydrolyzed polyacrylamide/Cr(III) systems.

[CR6] Dukhin AS (2007). Observation of sol–gel transition for carbon nanotubes using electroacoustics: colloid vibration current versus streaming vibration current. J Colloid Interface Sci.

[CR7] Huang Wen Lai, Liang Kai Ming, Cui Shi Hua (2001). Influence of ultrasonic vibration on the mesoporosity and surface fractal dimensions of resultant silica xerogels. Mater Res Bull.

[CR8] Junowicz E, Charm SE, Blair HE (1972). Gel filtration with vibration. Anal Biochem.

[CR9] Kang MJ, Pu CS (2007). Research and expectation of compound vibration stimulating technique. Oil Field Equip.

[CR10] Ma JG, Jin YH, Zhou SP (1996). Experiments on the effects of mechanical vibration on core permeability. J Xi’an Shiyou Univ (Natural Science Edition).

[CR11] Meynie L, Fenouillot F, Pascault J-P (2004). Influence of the gel on the morphology of a thermoset polymerized into a thermoplastic matrix, under shear. Polymer.

[CR12] Prud’homme RK, Princeton U, Uhl JT (1984) Kinetics of polymer/metal-ion gelation. SPE 12640-MS

[CR13] Walkenstrom P, Panighetti N, Windhab E (1998). Effects of fluid shear and temperature on whey protein gels, pure or mixed with xanthan. Food Hydrocoll.

[CR14] Wang HX, Zhang GY (2003). Effect of AgNO_3_ and As-produced Ag on rheological properties of hexagonal liquid crystal. Acta Chim Sin.

[CR15] Wang GX, Wang ZH, Chen ZQ (1995). Rate equation of gelation of chromium(III)-polyacrylamide sol. Chin J Chem.

[CR16] White JE (1975). Computed seismic speeds and attenuation in rocks with partial gas saturation. Geophysics.

[CR17] Zhang S, Wang YS, Zeng XT (2007). Evaluation of interfacial shear strength and residual stress of sol–gel derived fluoridated hydroxyapatite coatings on Ti6Al4V substrates. Eng Fract Mech.

